# Matrix metalloproteinase 9 induces endothelial-mesenchymal transition via Notch activation in human kidney glomerular endothelial cells

**DOI:** 10.1186/s12860-016-0101-0

**Published:** 2016-04-29

**Authors:** Ye Zhao, Xi Qiao, Lihua Wang, Tian Kui Tan, Hong Zhao, Yun Zhang, Jianlin Zhang, Padmashree Rao, Qi Cao, Yiping Wang, Ya Wang, Yuan Min Wang, Vincent W. S. Lee, Stephen I. Alexander, David C. H. Harris, Guoping Zheng

**Affiliations:** Centre for Transplant and Renal Research, Westmead Institute for Medical Research, the University of Sydney, 176 Hawkesbury Road, Sydney, NSW 2145 Australia; The School of Biomedical Sciences, Chengdu Medical College, Chengdu, 610500 PR China; Department of Nephrology, Second Hospital of Shanxi Medical University, Shanxi Kidney Disease Institute, WuYi Road 382, Taiyuan, 030001 Shanxi PR China; Department of Biochemistry and Molecular Biology, Shanxi Medical University, Xinjian Road 56, Taiyuan, 030001 Shanxi PR China; Experimental Centre of Science and Research, the First Clinical Hospital of Shanxi Medical University, Xinjian Road 382, Taiyuan, 030001 Shanxi PR China; Centre for Kidney Research, Children’s Hospital at Westmead, 212 Hawkesbury Road, Sydney, NSW Australia

**Keywords:** Matrix metalloproteinase 9, Endothelial-mesenchymal transition, Human glomerular endothelial cells, TGF-β1, Notch

## Abstract

**Background:**

Endothelial-mesenchymal transition (EndoMT) is a major source of myofibroblast formation in kidney fibrosis. Our previous study showed a profibrotic role for matrix metalloproteinase 9 (MMP-9) in kidney fibrosis via induction of epithelial-mesenchymal transition (EMT). Inhibition of MMP-9 activity reduced kidney fibrosis in murine unilateral ureteral obstruction. This study investigated whether MMP-9 also plays a role in EndoMT in human glomerular endothelial cells.

**Results:**

TGF-β1 (10 or 20 ng/ml) induced EndoMT in HKGECs as shown by morphological changes. In addition, VE-cadherin and CD31 were significantly downregulated, whereas α-SMA, vimentin, and N-cadherin were upregulated. RT-PCR revealed that Snail, a known inducer of EMT, was upregulated. The MMP inhibitor GM6001 abrogated TGF-β1-induced EndoMT. Zymography indicated that MMP-9 was also upregulated in TGF-β1-treated HKGECs. Recombinant MMP-9 (2 μg/ml) induced EndoMT in HKGECs via Notch signaling, as evidenced by increased formation of the Notch intracellular domain (NICD) and decreased Notch 1. Inhibition of MMP-9 activity by its inhibitor showed a dose-dependent response in preventing TGF-β1-induced α-SMA and NICD in HKGECs, whereas inhibition of Notch signaling by γ-secretase inhibitor (GSI) blocked rMMP-9-induced EndoMT.

**Conclusions:**

Taken together, our results demonstrate that MMP-9 plays an important role in TGF-β1-induced EndoMT via upregulation of Notch signaling in HKGECs.

## Background

Kidney fibrosis is an inevitable consequence of a wide variety of progressive chronic kidney diseases (CKD) that progress to end-stage kidney failure, a devastating disorder that requires kidney replacement therapies such as dialysis or kidney transplantation. Kidney fibrosis is characterized by tubular atrophy/dilation, interstitial leukocyte infiltration, fibroblast accumulation, and increased interstitial matrix deposition [1, 2]. Although different cells are involved in kidney fibrosis, fibroblasts or myofibroblasts are considered to play a pivotal role [3]. However, the cellular origins of myofibroblasts are diverse. Resident fibroblasts [4], fibrocytes from bone marrow [4, 5], pericytes and perivascular fibroblasts [5, 6], tubular epithelial cells [4, 5, 7], podocytes [8] and endothelial cells [7, 9] have been identified as contributing to the myofibroblast population.

Endothelial-mesenchymal transition (EndoMT) is process similar to that of epithelial-mesenchymal transition (EMT). During EndoMT, endothelial cell markers are downregulated whereas mesenchymal markers are upregulated. EndoMT is involved in organ development and various types of fibrosis. For example, EndoMT contributes to cardiac fibrosis [10–12], pulmonary fibrosis [10, 13], corneal fibrosis [14], radiation-induced pelvic disease [15] and inflammatory bowel disease-associated fibrosis [16]. EndoMT was also found in the early development of kidney interstitial fibrosis in the streptozocin (STZ)-induced diabetic nephropathy (DN) model [17, 18]. In addition, EndoMT also contributes to carcinoma-associated fibroblasts [19], atherogenesis, inflammation and hypertension [20, 21]. LeBleu et al. showed that ~10 % of the interstitial myofibroblasts co-stained with markers of endothelial cells and activated fibroblasts in unilateral ureteral obstruction (UUO) mice [7]. Zeisberg and colleagues [9] have demonstrated a role for EndoMT in several models of kidney disease. These studies demonstrated that activated fibroblasts co-express the endothelial marker CD31 as well as fibroblast markers such as fibroblast specific protein-1 (FSP-1) and α-smooth muscle actin (α-SMA). To demonstrate the presence of EndoMT-derived fibroblasts, these authors also used lineage tagged transgenic mice to trace endothelial lineage. Taken together, these findings suggest EndoMT plays a critical role in myofibroblast formation. EndoMT inhibition or reversal might be a potential target for treatment and prevention of kidney fibrosis.

Notch family is involved in podocyte and kidney tubular cell differentiation [22]. Abnormal Notch pathway activation can lead to glomerulonephritis (GN) and focal segmental glomerulosclerosis (FSGS) [23, 24]. Notch signaling is typically activated upon binding of ligands (such as Dll1, Dll3, Dll4, Jag1, and Jag2) with Notch 1–4 receptors. Then intramembrane proteolysis such as ectodomain shedding of both the ligand and the receptor, releasing the intracellular domains (ICD) of the ligand and receptor, thereby allowing Notch ICD (NICD) nuclear translocation to regulate gene expression.

We previously found that matrix metalloproteinase 9 (MMP-9) is capable of inducing tubular cell EMT and contribute to tubulointerstitial fibrosis [25, 26]. Although endothelial cells are capable of expressing MMP-9 [18], whether MMP-9 plays a role in EndoMT was unknown. In the current study, we defined a role for MMP-9 in EndoMT via Notch signaling.

## Methods

### Cell culture and treatment

Human kidney glomerular endothelial cells (HKGECs) were cultured in endothelial cell media (ScienCell; Carlsbad, CA, USA) containing vascular endothelial growth factor (VEGF; Sigma-Aldrich; St. Louis, MO, USA; 2.5–5 μg/ml) in basal medium (ScienCell), 5 % fetal bovine serum (FBS; ScienCell), 1 % endothelial cell growth supplement (ECGS; ScienCell) and 1 % penicillin/streptomycin (P/S; ScienCell). Cells were maintained at 37 °C with 5 % CO_2_. For treatment, HKGECs were cultured for 24 h at low density in flasks or plates pre-coated with fibronectin and washed in PBS. Cells were treated with TGF-β1 (10 ng/ml or 20 ng/ml; Sigma-Aldrich) alone, TGF-β1 plus 10 μM GM6001 (Calbiochem; Darmstadt, Germany), TGF-β1 plus the MMP-9 inhibitor I (Merck Chemicals; Darmstadt, Germany) at different dosages (0.05 nmol/ml, 0.25 nmol/ml, and 0.5 nmol/ml), recombinant MMP-9 (rMMP-9; 2 μg/ml; Biomol International; Plymouth Meeting, PA, USA), or rMMP-9 (2 μg/ml) plus the gamma secretase inhibitor (GSI; 5–10 μM; Merck Millipore; Billerica, MA, USA). Our study does not require any human or animal ethics approval.

### Immunofluorescence analysis

For indirect immunofluorescence, HKGECs were cultured on glass coverslips, washed twice in PBS, fixed with absolute methanol for ten minutes at −20 °C, and blocked for 1 h with 2 % BSA (Sigma) at room temperature. Cells were then incubated for 1 h at room temperature with primary antibodies against endothelial markers rabbit polyclonal anti-VE-cadherin (1:200; Alexis Biochemicals; Farmingdale, NY, USA), mouse monoclonal anti-CD31 (1:100; Cell Signaling Technology; Boston, MA, USA) and mesenchymal markers mouse monoclonal anti-α-SMA (1:200; Sigma Chemical Co), rabbit monoclonal anti-vimentin (1:200; Cell Signaling Technology) and rabbit monoclonal anti-N-cadherin (1:100, BD Bioscience; San Jose, CA, USA) in 2 % BSA. The following secondary antibodies were used: goat anti-mouse IgG2a/2b phycoerythrin (PE)-conjugated antibody (1:400; Invitrogen; Carlsbad, CA, USA) for CD31, α-SMA and goat anti-rabbit IgG2a/2b FITC-conjugated antibody (1:400; Invitrogen) for VE-cadherin, vimentin and N-cadherin. Cells were washed twice with PBS, counterstained with DAPI for 5 min, and washed twice with PBS. Samples were mounted using fluorescence mounting media. For negative isotype controls staining, rat IgG2a κ purified (eBioscience; San Diego, CA, USA) was used for VE-cadherin, vimentin and N-cadherin mouse IgG2a κ (Biolegend; San Diego, CA, USA) was used for CD31 and α-SMA, and their corresponding secondary antibodies were applied.

### RNA extraction, purification, and quantitation

Total RNA was extracted from cultured cells using 350 μl of RLT buffer and homogenized by shredding through a 0.5 ml insulin syringe 5 times. Extracted RNA was purified using RNeasy Mini Kit (Qiagen; Hilden, Germany) following the manufacturer’s instruction and resuspended in 30 μl of RNAse-free water. The yield and purity of RNA was measured spectrophotometrically by absorption at 260 nm (A260) and 280 nm (A280) using a Beckman-Coulter DU800 spectrophotometer (CA, USA).

### Real-time RT-PCR analysis

cDNAs were synthesized using 200 ng of extracted RNA in 20 μl reaction buffer by reverse transcription using Superscript^TM^ First Strand Synthesis System (Invitrogen) and random hexamer primers at 50 °C for 50 min. Designed primers and established primers from published papers were used for Real-time RT-PCR. The sequences of hes-1 primers used for this analysis are as follows: forward: 5′-GAC AGC ATC TGA GCA CAG AAA TG-3′ and reverse: 5′- GTC ATG GCA TTG ATC TGG GTC AT-3′ [27]. Housekeeping gene β-actin was used as the internal control. For Real-time RT-PCR, PCR mixture contained 0.5 μl of cDNA and 10 ρmol/μl of each primer in a 20 μl final volume of SYBR mastermix (Invitrogen). Amplification was performed using the Rotogene-6000 Real-Time cycler thermos.

### Western blot analysis

Equal volumes or quantities of protein were loaded in 12-well NuPAGE 4–15 % Bis-Tris gels (Bio-Rad; Hercules, CA, USA) and electrophoresed under reducing conditions. After electrophoresis, proteins were transferred for 2 h to PVDF membranes using a Mini Trans-Blot Electrophoretic Transfer Cell apparatus (Bio-Rad). For immunodetection, membranes were blocked overnight at 4 °C in 5 % skim milk and incubated for 2 h at room temperature with the primary antibody against mouse monoclonal anti-α-SMA (1:300, Sigma Chemical Co.), rabbit polyclonal anti-VE-cadherin (1:1000; Alexis Biochemicals), mouse monoclonal anti-CD31 (1:1000; Cell Signaling Technology), mouse polyclonal anti-NICD (1;1000; Merck Millipore, Cat. # 07–1232) [28], β-actin (1:3000, Sigma), rabbit monoclonal anti-Notch1 (1:1000; Cell Signaling Technology), rabbit monoclonal anti-vimentin (1:1000; Cell Signaling Technology), rabbit monoclonal anti-N-cadherin (1:500, BD BioSciences), rabbit polyclonal anti-Hes-1 (1:500, Abcam; Cambridge, UK) and rabbit polyclonal anti-Hey-1 (1:500, Abcam) prepared in blocking buffer. Membranes were washed three times (10 min per wash on a rocking platform) and incubated for 1 h with their respective horseradish peroxidase (HRP)-conjugated secondary antibodies; goat anti mouse HRP and goat anti-rabbit HRP (1:5000; Cell Signaling Technology) prepared in blocking buffer. Membranes were again washed three times (10 min per wash on a rocking platform). Bands were visualized using an enhanced chemiluminescence detection kit.

### Zymography

MMP-9 activity in medium derived from TGF-β treated HKGECs was determined by gelatin zymography. Briefly, medium was mixed with Tris-Glycine SDS Native Sample Buffer (1:1; Invitrogen) and electrophoresed through 10 % Novex Zymogram Gelatin Gels (Invitrogen) with Tris-Glycine SDS Running Buffer (Invitrogen) under constant voltage of 125 V for 120 min. After electrophoresis, gels was incubated in Zymogram Renaturing Buffer (Invitrogen) for 30 min at room temperature with gentle agitation and washed with developing buffer (Invitrogen) for 30 min. The gel was further incubated for 24 h in fresh developing buffer at 37 °C. After developing, the gel was stained with 0.5 % (w/v) Coomassie Blue R-250 (Bio-Rad) in 50 % (v/v) methanol, 10 % (v/v) acetic acid for 30 min at room temperature, and destained as described previously [25]. Gelatinolytic activity of MMP-9 was visualized as a clear band on a blue background. Band intensity was quantified by densitometry using ImageJ software. Briefly, zymogram gels were scanned using Kodak gel logic 100 imaging system and processed into gray scale. Gray scale images were quantified densitometrically by the measurement of the mean intensity of positive band multiplied by its corresponding area. The optical band intensity was then corrected by subtracting background intensity of equal area.

### Statistical analysis

Results from at least three independent experiments are expressed as mean ± SEM. Statistical significance was evaluated using two-tail *t*-test for comparison between two groups, whereas the one way analysis of variance (ANOVA) was used for comparison of multiple groups. *P <* 0.05 was considered significant.

## Results

### TGF-β1 induces EndoMT in HKGECs

**Fig. 1:**
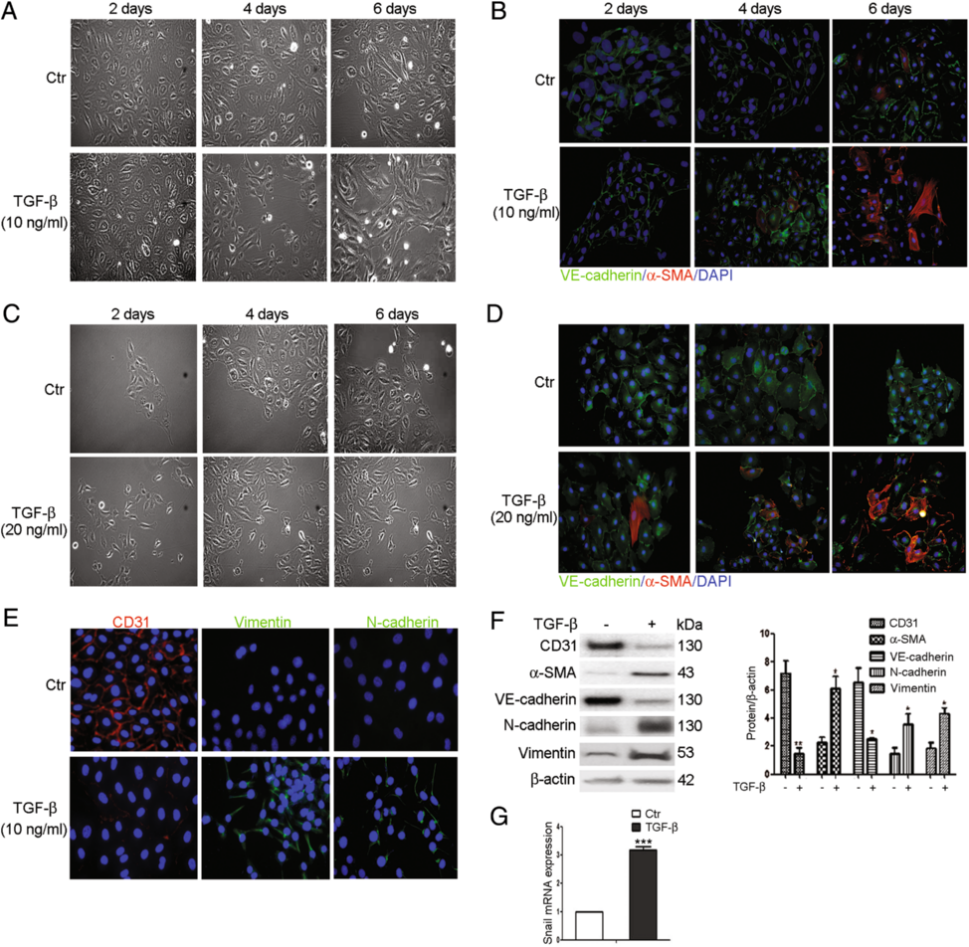
TGF-β1 induces EndoMT in HKGECs. **a** Morphological changes in HKGECs induced by TGF-β1 (10 ng/ml) were examined using phase contrast microscopy. **b** Indirect immunofluorescence staining and co-localization of VE-cadherin and α-SMA were performed in HKGECs cultured in Endothelial Cell Medium with TGF-β1 (10 ng/ml). **c** Morphological changes in HKGECs induced by TGF-β1 (20 ng/ml) were examined using phase contrast microscopy. Cells were counterstained with DAPI to visualize nuclei (*blue*). **d** Indirect immunofluorescence staining and co-localization of VE-cadherin and α-SMA were performed in HKGECs cultured in Endothelial Cell Medium with TGF-β1 (20 ng/ml). **e** Indirect immunofluorescence staining of CD31, vimentin, and N-cadherin was performed in HKGECs cultured in Endothelial Cell Medium with TGF-β1 (10 ng/ml). **f** Respective western blot analysis and quantitation of CD31, α-SMA, VE-cadherin, N-cadherin, and vimentin in HKGECs treated with TGF-β1 (10 ng/ml). β-actin was used as a loading control. **g** Snail mRNA expression from HKGECs cultured in Endothelial Cell Medium with TGF-β1 (10 ng/ml) was quantified using real-time PCR. Gene expression levels were normalized to human GADPH mRNA. Original magnification × 200. Data are expressed as mean ± SEM with *n* ≥ 3 for each experimental group, *P < 0.05, **P < 0.01. Morphological and immunofluorescent are representative of experiments repeated 3 times

To determine whether TGF-β induces EndoMT, HKGECs were treated in the presence or absence of TGF-β1. HKGECs exhibited morphological changes typical of EndoMT, as shown by phenotypic transformation from an endothelial cobblestone shape to fibroblastic spindle-shaped morphology by day 2 (Fig. 1a) and increasing numbers of fibroblasts evident on days 4 to 6 (Fig. 1a). Cells cultured in the absence of TGF-β showed fewer morphological changes. The transition from an endothelial to mesenchymal phenotype was confirmed using immunofluorescent staining. Endothelial cells treated with TGF-β1 (10 and 20 ng/ml) lost VE-cadherin expression and acquired α-SMA expression (Fig. 1b and c). When treated with higher concentration of TGF-β1 (20 ng/ml), morphological changes in HKGECs typical of EndoMT was also induced (Fig. 1c). Immunofluorescence staining showed decreased expression of VE-cadherin and increased expression of α-SMA, particularly in samples that were treated with TGF-β1 for 6 days (Fig. 1d). However, 20 ng/ml TGF-β1 could not induce EndoMT in a shorter time. There were no notable differences in α-SMA-stained cells at days 2 and 4 compared to 10 ng/ml TGF-β1 treatments. These results suggest that TGF-β1 is capable of inducing EndoMT in HKGECs in 6 days. To further confirm TGF-β induction of EndoMT in HKGECs, cells were stained with the endothelial marker CD31 and the mesenchymal markers vimentin and N-cadherin. The transition from an endothelial phenotype to a mesenchymal phenotype was confirmed using immunofluorescent staining. Upon TGF-β1 treatment, endothelial cells lost CD31 expression and acquired vimentin and N-cadherin expression (Fig. 1e). Immunofluorescent staining specificity was confirmed by negative control staining for each isotype control antibody. Consistent with the immunofluorescence staining results, western blot analysis revealed decreased levels of the endothelial markers VE-cadherin and CD31 in HKGECs treated with TGF-β1. However, there was increased expression of the mesenchymal markers α-SMA, vimentin, and N-cadherin (Fig. 1f). EndoMT was also confirmed by real-time PCR analysis. We observed upregulation of the transcription factor Snail mRNA in HKGECs treated with TGF-β1 compared to control HKGECs (Fig. 1g). Taken together, these results demonstrate that TGF-β1 induces EndoMT in HKGECs.

### The MMP inhibitor GM6001 inhibits TGF-β1-induced EndoMT in HKGECs

**Fig. 2:**
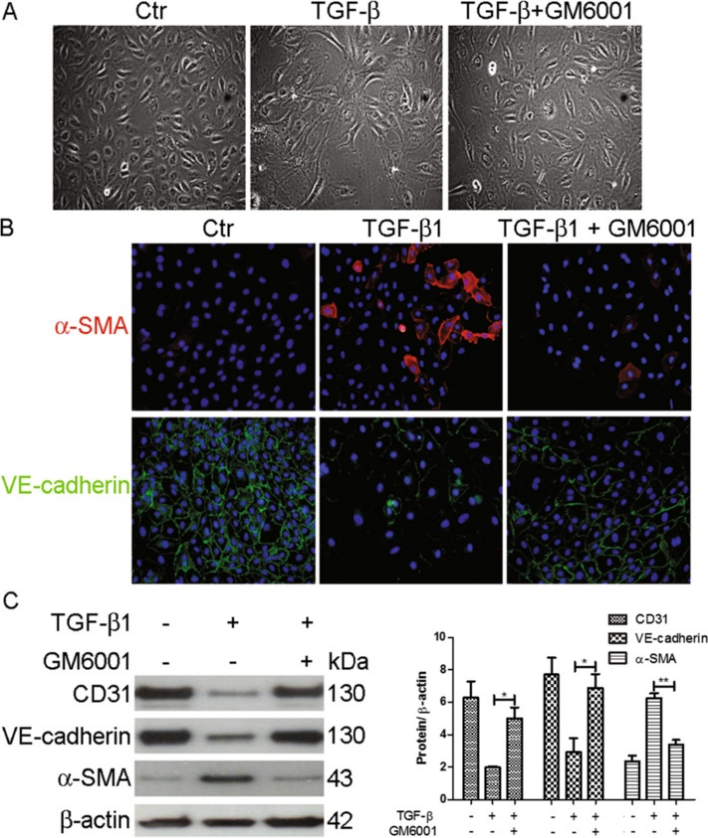
GM6001 inhibits TGF-β1-induced EndoMT in HKGECs. **a** Morphological changes in HKGECs induced by TGF-β1 (10 ng/ml) in the presence or absence of GM6001 were examined using phase contrast microscopy. **b** Indirect immunofluorescence staining of VE-cadherin and α-SMA were performed in HKGECs cultured in Endothelial Cell Medium with TGF-β1 (10 ng/ml). **c** Respective western blot analysis and quantitation of CD31, VE-cadherin and α-SMA were performed in HKGECs treated with TGF-β1 (10 ng/ml) in the presence or absence of GM6001. β-actin was used as a loading control. Original magnification × 200. Image are representative and data are expressed as mean ± SEM with *n* ≥ 3 for each experimental group, *P < 0.05, **P < 0.01

To determine whether MMPs contribute to TGF-β1-induced EndoMT, HKGECs were treated with the broad-spectrum MMP inhibitor GM6001. After treatment for 6 days, we found that EndoMT induced by 10 ng/ml TGF-β1 was abrogated by GM6001; the majority of HKGECs maintained typical endothelial cobblestone morphology with few cells exhibiting fibroblastic spindle-shape morphology (Fig. 2a). Consistent with the cellular morphology, immunofluorescence (Fig. 2b) and western blot analysis (Fig. 2c) indicated that GM6001 abrogated the TGF-β1-induced decrease in CD31 and VE-cadherin expression and increase in α-SMA expression. Collectively, these results suggest that MMPs are involved in TGF-β1-induced EndoMT in HKGECs.

### MMP-9 is involved in TGF-β1-induced EndoMT in HKGECs

**Fig. 3:**
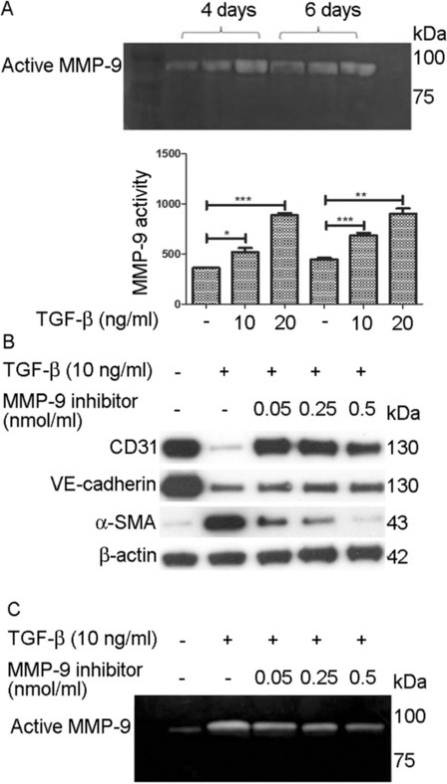
Evaluation of MMP-9 activity. **a** MMP-9 activity in response to TGF-β was examined using gelatin zymography. Relative activity was compared to control (without TGF-β treatment). **b** CD31, VE-cadherin, and α-SMA expression in HKGECs treated with TGF-β1 (10 ng/ml) and various dosages of the MMP-9 inhibitor (0.05, 0.25, and 0.5 nmol/ml) were evaluated using western blot analysis. β-actin was used as a loading control. **c** MMP-9 activity was determined using gelatin zymography. Relative activity was compared to control (without TGF-β1 treatment). Data are expressed as mean ± SEM with *n* ≥ 3 for each experimental group, *P < 0.05, **P < 0.01, ***P < 0.001

Gelatin zymography showed that MMP-9 activity increased significantly within 4 days in the presence of conditioned media with 10 ng/ml or 20 ng/ml TGF-β1 (Fig. 3a). After the cells had been cultured for 6 days, MMP-9 activity in the media was upregulated in both the presence of 10 ng/ml or 20 ng/ml TGF-β1. No MMP-2 expression was detected in the media (Fig. 3a).

To further examine whether MMP-9 is involved in EndoMT, HKGECs were treated with TGF-β1 in presence of MMP-9 inhibitor. Our results demonstrated that the MMP-9 inhibitor reduced α-SMA expression in the TGF-β1-treated samples. In addition, when different dosages of MMP-9 inhibitor were used, the reduction in α-SMA and recovery of VE-cadherin expression was dose dependent (Fig. 3b). To confirm that the MMP-9 inhibitor was functional, zymography was performed to evaluate MMP-9 and MMP-2 activity. The results showed that the MMP-9 inhibitor successfully inhibited MMP-9 activity (Fig. 3c).

### rMMP-9 induces EndoMT in HKGECs

**Fig. 4:**
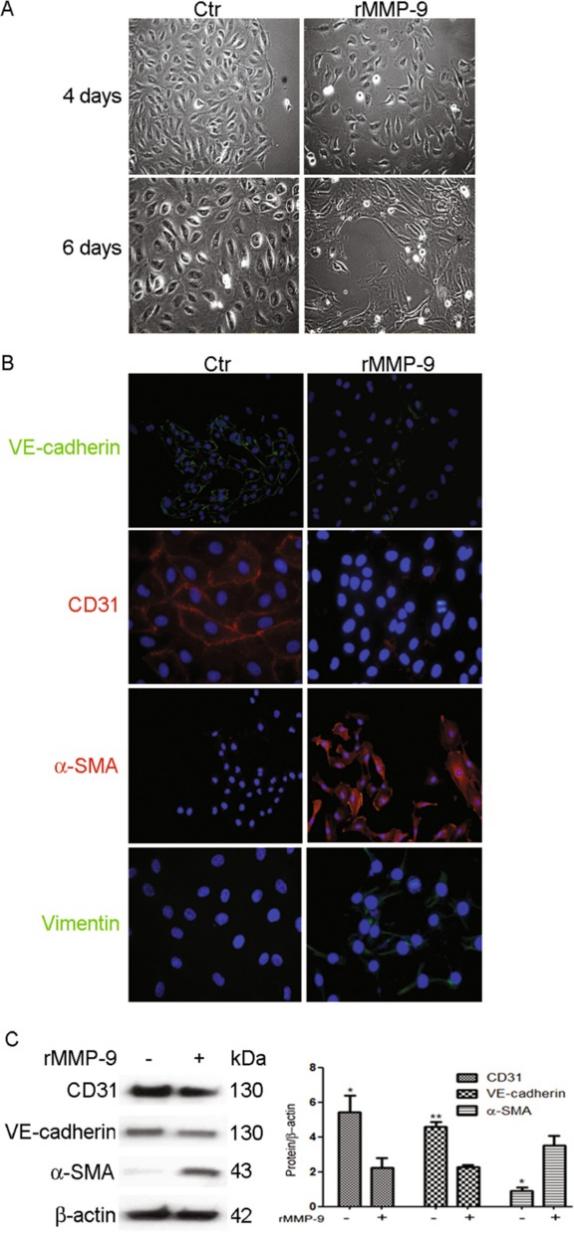
rMMP-9 induces EndoMT in HKGECs. **a** rMMP-9-induced morphological changes in HKGECs were examined using phase contrast microscopy. **b** Indirect immunofluorescence staining of CD31, α-SMA, VE-cadherin, and vimentin was performed in HKGECs cultured in Endothelial Cell Medium in the presence or absence of rMMP-9 (2 μg/ml). **c** Western blot analysis of CD31, VE-cadherin and α-SMA in HKGECs treated or not with rMMP-9 (2 μg/ml). β-actin was used as a loading control. Original magnification × 200. Data are expressed as mean ± SEM with *n* ≥ 3 for each experimental group, *P < 0.05, **P < 0.01

To determine whether MMP-9 contributes to EndoMT, subconfluent HKGECs were treated with rMMP-9 (2 μg/ml). rMMP-9 induced HKGEC phenotypic transformation from the endothelial cobblestone shape to the fibroblastic spindle-shaped morphology within 6 days of treatment (Fig. 4a). The transition of an endothelial to mesenchymal phenotype was confirmed by immunofluorescence (Fig. 4b). Endothelial cells lost VE-cadherin and CD31 expression and acquired α-SMA and vimentin expression. In addition, VE-cadherin, CD31, and α-SMA expression was examined by western blot analysis (Fig. 4c). Consistent with the immunofluorescence results, western blot analysis revealed that in HKGECs treated with rMMP-9, levels of the endothelial markers VE-cadherin and CD31 decreased, whereas the mesenchymal marker α-SMA increased (Fig. 4c). Taken together, these results demonstrate that rMMP-9 induces EndoMT in HKGECs.

### Notch signaling is activated in TGF-β1-induced EndoMT but is inhibited by MMP-9 inhibitor

**Fig. 5:**
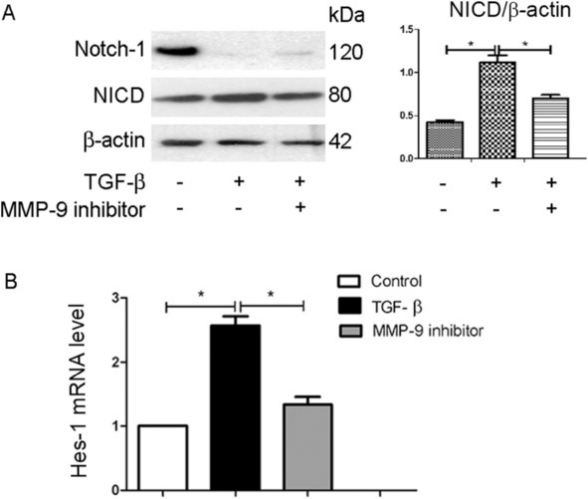
The Notch pathway is activated in TGF-β1-induced EndoMT. **a** Respective western blot analysis and quantitation of Notch-1 and NICD expression in TGF-β1 (10 ng/ml) induced EndoMT in HKGECs six days after treatment. β-actin was used as a loading control. **b** qPCR analysis of Hes-1 expression in TGF-β1-induced EndoMT in HKGECs. Data are expressed as mean ± SEM with *n* ≥ 3 for each experimental group, *P < 0.05, **P < 0.01

To investigate Notch signaling in TGF-β1-induced EndoMT, we examined the expression of Notch 1 protein and Notch intracellular domain (NICD) using western blot analysis. We also evaluated the Notch downstream transcriptional factors Hes-1 and Hey-1 using qPCR. We found that when HKGECs were treated with TGF-β1, Notch-1 expression was significantly down-regulated whereas NICD was significantly increased compared to control (Fig. 5a). In addition, when TGF-β1-induced cells were treated with the MMP-9 inhibitor, NICD expression decreased (Fig. 5a). The qPCR results showed that TGF-β1-induced Hes-1 expression in HKGECs was significantly reduced by MMP-9 inhibitor (Fig. 5b). These results show that MMP-9 contributes to the Notch pathway activation in TGF-β1-induced EndoMT.

### rMMP-9 activates Notch signaling

**Fig. 6:**
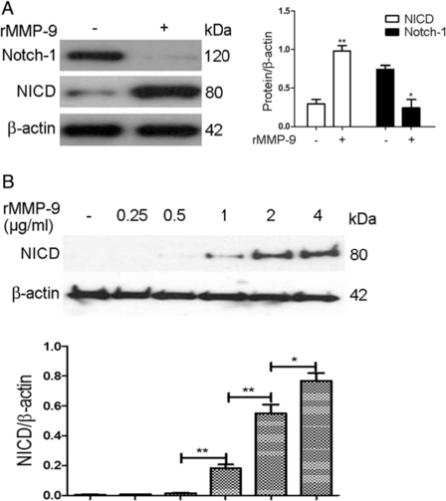
The Notch pathway is activated in rMMP-9-induced EndoMT. **a** Respective Western blot analysis and quantitation of Notch-1 and NICD expression in rMMP-9-induced EndoMT in HKGECs six days after treatment. **b** Western blot analysis and quantitation showing NICD expression is regulated in an rMMP-9 dose-dependent manner. β-actin was used as a loading control. Data are expressed as mean ± SEM with *n* ≥ 3 for each experimental group, *P < 0.05, **P < 0.01

We also examined Notch pathway activation in rMMP-9-induced HKGECs using western blot analysis. After HKGECs were incubated with rMMP-9, the Notch-1 protein was cleaved and NICD was released (Fig. 6a). To examine whether rMMP-9 can activate Notch signaling, different dosages of rMMP-9 (0, 0.25, 0.5, 1.0, 2.0, and 4.0 μg/ml) were added to HKGECs. We found that NICD level was increased in a rMMP-9 dose-dependent manner (Fig. 6b).

### Inhibition of Notch signaling by γ-secretase inhibitor (GSI) blocks rMMP-9-induced EndoMT

**Fig. 7:**
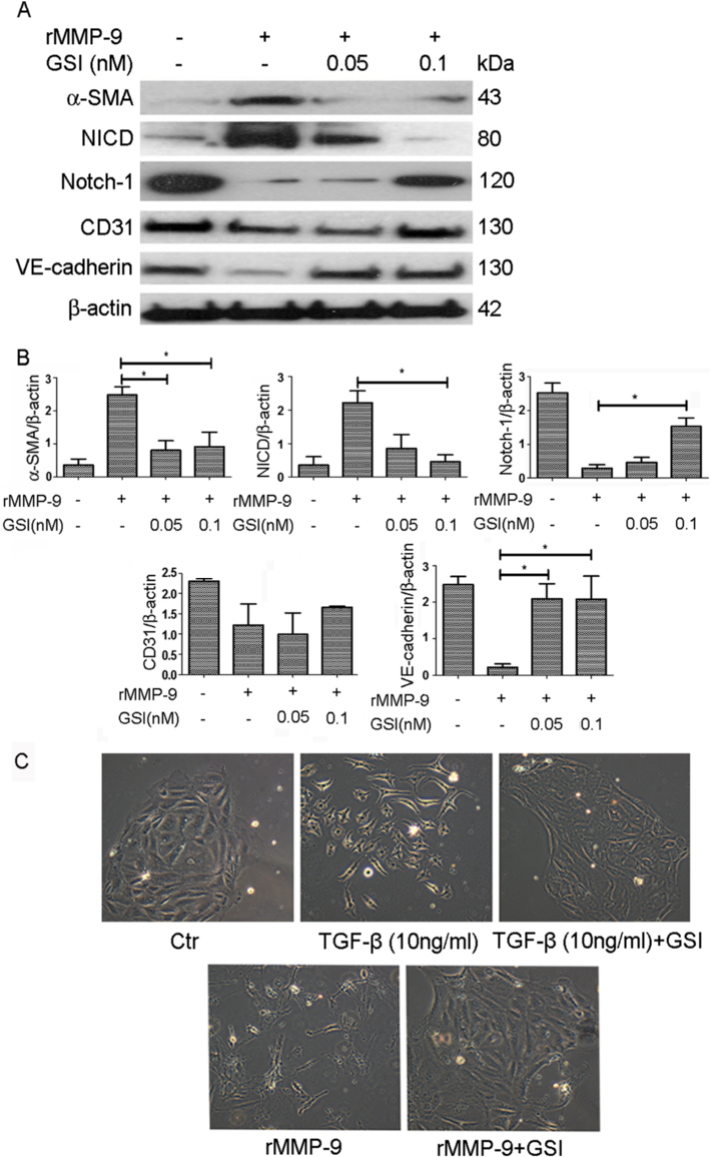
The γ-secretase inhibitor GSI-I regulates protein expression in rMMP-9-induced EndoMT. **a** Western blot analysis of α-SMA, NICD, Notch-1, CD31 and VE-cadherin expression in rMMP-9-induced EndoMT in the presence or absence of GSI-I six days after treatment. β-actin was used as a loading control. **b** Statistical analysis of protein expression in rMMP-9-induced EndoMT in the presence or absence of GSI-I. **c** Morphological changes in HKGECs induced by TGF-β1 (10 ng/ml) or rMMP-9 in the presence or absence of GSI. Data are expressed as mean ± SEM with *n* ≥ 3 for each experimental group, *P < 0.05

Notch signaling was inhibited using GSI, an effective γ-secretase inhibitor (Z-Leu-Leu-Nle-CHO) [29]. Western blot results (Fig. 7a) and statistical analysis (Fig. 7b) showed that GSI significantly reduced α-SMA and NICD expression and increased CD31, VE-cadherin and Notch-1 expression of cells exposed to MMP-9 compared to cells treated with rMMP-9 alone. GSI inhibited both TGF-β1- and MMP-9-induced morphological changes (Fig. 7c). This result demonstrates that the rMMP-9-induced EndoMT is Notch signaling dependent.

## Discussion

Kidney EndoMT is a major source of fibroblast formation in kidney fibrosis, and has emerged as a potentially important mechanism in development and progression of kidney fibrosis [9]. In a recent study, LeBleu et al. found that endothelial cells play a more important role than epithelial cells and pericytes in myofibroblast formation via EndoMT [7]. Therefore, understanding and halting EndoMT is an important clinical challenge.

Kidney endothelial cells consist of glomerular, vascular and peritubular endothelial cells. Although it has been generally accepted that endothelial cells contribute to fibroblast formation in kidney, whether glomerular endothelial cells contribute to fibroblast formation remains unclear. In our recent study, we showed that mice peritubular endothelial cells have an important role in kidney EndoMT. Moreover, we found that MMP-9 was involved in peritubular EndoMT, likely via the Notch pathway. Human glomerular endothelial cells are likely to be another instructive model for studying kidney fibrosis.

Here, we demonstrate that TGF-β1 and rMMP-9 induced EndoMT in HKGECs via Notch signaling. The Notch signaling pathway is a highly conserved cascade in mammals that regulates multiple cellular processes, including proliferation, differentiation, and apoptosis [30, 31]. Notch signaling has been shown to be downstream of VEGF to regulate endothelial cell morphogenesis [32]. In addition, Notch activation induces endothelial cell morphological, phenotypic, and functional changes consistent with mesenchymal transformation [33]. Notch signaling has been found to initiate EndoMT in atrioventricular endothelial cells [34]. Inhibition of Notch signaling ameliorates EMT and tubulointerstitial fibrosis in mouse [35]. Importantly, Notch inhibition reversed podocyte injury (EMT) and kidney failure [36]. All these findings suggest a critical role for Notch signaling in both EMT and EndoMT which are pivotal processes in kidney fibrosis. Our results demonstrate that MMP-9 activation of Notch signaling in glomerular endothelial cells is downstream of TGF-β1. Combining with observations with previous studies from our laboratory and others, a strong profibrotic role for MMP-9 is evident. It promotes EMT of tubular epithelial cells, EndoMT of peritubular endothelial cells and EndoMT of glomerular endothelial cells, thereby, leading to kidney fibrosis in both tubulointerstial and glomeruli compartments.

## Conclusion

Our data demonstrate that MMP-9 plays an important role in TGF-β1-induced EndoMT in HKGECs, via upregulation of Notch signaling. Thus, inhibition of MMP-9 or Notch signaling could be therapeutic strategies for treatment for kidney fibrosis in CKD.

## Abbreviations

ANOVA: one way analysis of variance
CKD: chronic kidney diseases
DN: diabetic nephropathy
ECGS: endothelial cell growth supplement
EMT: epithelial-mesenchymal transition
EndoMT: endothelial-mesenchymal transition
FBS: fetal bovine serum
FSGS: focal segmental glomerulosclerosis
FSP-1: fibroblast specific protein-1
GN: glomerulonephritis
GSI: gamma secretase inhibitor
HRP: horseradish peroxidase
ICD: intracellular domains
MMP-9: matrix metalloproteinase 9
NICD: Notch intracellular domains
P/S: penicillin/streptomycin
PE: phycoerythrin
TGF-β1: transforming growth factor-beta1
UUO: unilateral ureteral obstruction
VEGF: vascular endothelial growth factor
α-SMA: α-smooth muscle actin
