# Correction to: Matrix metalloproteinase 9 induces endothelial-mesenchymal transition via Notch activation in human kidney glomerular endothelial cells

**DOI:** 10.1186/s12860-020-00318-6

**Published:** 2020-10-21

**Authors:** Ye Zhao, Xi Qiao, Lihua Wang, Tian Kui Tan, Hong Zhao, Yun Zhang, Jianlin Zhang, Padmashree Rao, Qi Cao, Yiping Wang, Ya Wang, Yuan Min Wang, Vincent W. S. Lee, Stephen I. Alexander, David C. H. Harris, Guoping Zheng

**Affiliations:** 1 Centre for Transplant and Renal Research, Westmead Institute for Medical Research, the University of Sydney, 176 Hawkesbury Road, Sydney, NSW 2145 Australia; 2 The School of Biomedical Sciences, Chengdu Medical College, Chengdu, 610500 PR China; 3 Department of Nephrology, Second Hospital of Shanxi Medical University, Shanxi Kidney Disease Institute, WuYi Road 382, Taiyuan, 030001 Shanxi PR China; 4 Department of Biochemistry and Molecular Biology, Shanxi Medical University, Xinjian Road 56, Taiyuan, 030001 Shanxi PR China; 5 Experimental Centre of Science and Research, the First Clinical Hospital of Shanxi Medical University, Xinjian Road 382, Taiyuan, 030001 Shanxi PR China; 6 Centre for Kidney Research, Children’s Hospital at Westmead, 212 Hawkesbury Road, Sydney, NSW Australia

**Correction to: BMC Cell Biol 17, 21 (2016)**

**https://doi.org/10.1186/s12860-016-0101-0**

Following publication of the original article [1], an error was reported in Fig. 1c. In the TGF-β (20 ng/ml) row, the “4 days” and “6 days” panels were accidentally duplicated. The correct Fig. 1c with the correct “6 days” panel is given below.

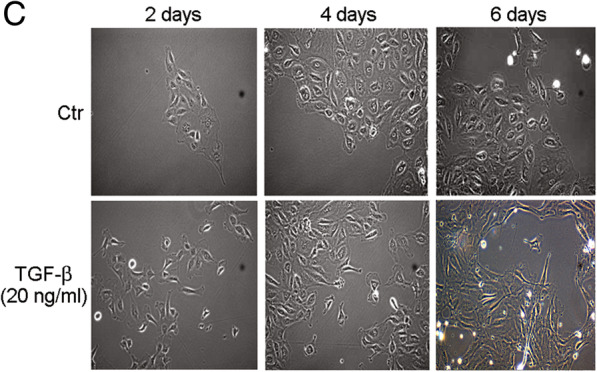



Fig. 1 **c** Morphological changes in HKGECs induced by TGF-β1 (20 ng/ml) were examined using phase contrast microscopy. Cells were counterstained with DAPI to visualize nuclei (*blue*).
